# New Orleans’ school meals distribution in response to the COVID-19 pandemic: operational details and geographic coverage

**DOI:** 10.3389/fpubh.2023.1191325

**Published:** 2023-06-23

**Authors:** M. Pia Chaparro, Jacob French, Kristine Creveling, Naana Ennin, Tiffany Numa, Megan Knapp

**Affiliations:** ^1^Department of Social, Behavioral and Population Sciences, School of Public Health and Tropical Medicine, Tulane University, New Orleans, LA, United States; ^2^Guilford County Division of Public Health, Greensboro, NC, United States; ^3^Propeller: A Force for Social Innovation, New Orleans, LA, United States; ^4^Department of Public Health Sciences, Xavier University of Louisiana, New Orleans, LA, United States

**Keywords:** COVID-19, school meals, New Orleans, social vulnerability, nutrition

## Abstract

**Introduction:**

To facilitate continuation of school feeding during COVID-19 school lockdowns, U.S. Congress authorized waivers to allow for school meals to be picked up by parents/guardians in non-school settings. We summarized school meals distribution and characterized reach in socially vulnerable neighborhoods in New Orleans, a city prone to environmental disasters, with a city-wide charter school system, and historically high levels of child poverty and food insecurity.

**Methods:**

School meals operations data were obtained from New Orleans, Louisiana (NOLA) Public Schools for 3/16/2020–5/31/2020. For each pick-up location, we estimated: average meals available (weekly), average meals served (weekly), number of weeks of operation, and rate of meal pick-up ([meals served/meals available]*100). These characteristics were mapped in QGIS v3.28.3, along with neighborhoods’ Social Vulnerability Index (SVI). Pearson correlation and ANOVA were run to assess differences between operations characteristics and neighborhood SVI.

**Results:**

From 38 meal sites, 884,929 meals were available for pick-up; 74% of pick-up sites were in moderately/highly socially vulnerable areas. Correlations between average meals available and served, weeks of operation, rate of meal pick-up, and SVI were weak and not statistically significant. SVI was associated with average rate of meal pick-up but not other operations characteristics.

**Discussion:**

Despite the disaggregated nature of the charter school system, NOLA Public Schools successfully pivoted to providing children with pick-up meals due to COVID-19 lockdowns, with 74% of sites located in socially vulnerable neighborhoods. Future studies should describe the types of meals provided to students during COVID-19, in terms of diet quality and nutrient adequacy.

## 1. Introduction

The COVID-19 pandemic brought disruption to daily life, with mandated lockdowns implemented to slow down the spread of the virus leading to closures of businesses and schools. With reported increases in food insecurity nationwide (1, 2), the United States (U.S.) Congress responded by enacting the Families First Coronavirus Response Act (FFCRA) on March 18, 2020 (3). Among other things, FFCRA authorized meal waivers for child nutrition programs, including the National School Lunch Program and the School Breakfast Program, which provide free or reduced-price meals to children living in low-income households in the U.S. (4, 5). The FFCRA waivers included the ability for parents/guardians to pick-up meals for their children without them being present, the ability to serve meals outside regular mealtime, and the ability to serve meals in a non-congregate setting, which effectively allowed free meals to be picked up curbside for all youth <18 years regardless of where they attend school (6). This temporarily transformed the child nutrition programs which traditionally targeted children living in low-income households into a free school meal program for all.

This Brief Research Report summarizes the school meal distribution program that operated in New Orleans, Louisiana in response to the COVID-19 pandemic, including an evaluation of the geographic coverage of the program with respect to the social characteristics of the neighborhoods where meal distribution sites were placed. Unlike most U.S. cities with traditional schools with centralized governance through districts, New Orleans has a decentralized city-wide charter school system that serves 99% of the city’s public-school students (7). Charter schools are autonomously operated and set their own policies through nonprofit community boards. Families apply to send their children to schools of their choice, and enrollment is not based on residence.

New Orleans has among the highest rates of child poverty (8) and child food insecurity (9) in the nation. In 2019, the prevalence of child food insecurity in Orleans Parish (equivalent to the city of New Orleans) was 25.6%, compared to 14.6% for the U.S. as a whole (8) and, in 2018, Orleans Parish was among the five counties in the country ranking in the top 10% for food insecurity rates and high meal costs (10). Further, New Orleans is segregated by race and socioeconomic status, with Black households overrepresented in the poorest neighborhoods (11). Thus, it is crucial to evaluate if the COVID-19 school meal distribution program implemented in New Orleans was accessible to the city’s most vulnerable children given that the system of disaggregated school governances and non-guaranteed proximity of family residences to schools led to unique challenges in continuing school feeding operations in the immediate aftermath of COVID-19 related school closures.

## 2. Methods

Data on meal distribution operations related to the COVID-19 pandemic between March 16 and May 31, 2020 were obtained from NOLA Public Schools, the Orleans Parish’s school board. NOLA Public Schools oversees operations for 76 public schools in Greater New Orleans, including 6 kindergarten through 12th grade (K-12) schools, 48 elementary schools, and 22 high schools, having served 45,022 students in the 2020–2021 academic year, 82% of whom are Black (12). Data shared with the authors included the locations where school meals were available for pick-up (i.e., site address, including for schools, businesses, parking lots, churches, and other sites chosen based on the ability to serve large populations within walking distance); the days of operation for each of the locations; and the number of meals available and picked-up in each location for every date of operation. NOLA Public Schools resumed in-person schooling in August 2020; hence, this study is focused on the portion of the 2019–2020 academic year interrupted by COVID-19 closures.

At the start of the COVID-19 meal distribution program on March 16, 2020, meals were available for pick-up daily; however, as of March 30, 2020, meal pick-up became available twice per week on Mondays (two breakfasts and two lunches available per child to cover Monday and Tuesday) and Wednesdays (three breakfasts and three lunches available per child to cover Wednesday, Thursday, and Friday). Thus, school meal data were summarized based on weekly numbers to account for differences in daily vs. twice-weekly distributions.

Geocoded data from the American Community Survey (ACS) 2015–2019 (13) obtained from the National Historical Geographic Information System (NHGIS) (14) were used to categorize Orleans Parish’s census tracts based on their Social Vulnerability Index (SVI), with social vulnerability defined as the “socioeconomic and demographic factors that affect the resilience of communities” (15). The domains that form the basis of SVI are 1) *socioeconomic status*, including poverty, unemployment, income, and education, 2) *household composition/disability*, comprising of age, single parenting, and disability, 3) *minority status/language*, comprising of race, ethnicity, and English-language proficiency, and 4) *housing/transportation*, comprising of housing structure, crowding, and vehicle access (16). Variables extracted from ACS at the census tract level to construct the SVI following the domains above include: 1) number of individuals with an income-to-poverty ratio = 1 (i.e., living <100% federal poverty level [FPL]), 2) number of individuals in the civilian labor force who are unemployed, 3) *per capita* income, 4) number of individuals 25 years or older without a high school diploma, 5) number of individuals 65 years or older, 6) number of individuals 17 years or younger, 7) number of individuals with a disability, 8) number of households led by a single parent, 9) number of individuals who are not non-Hispanic White, 10) number of individuals who speak English “less than well,” 11) number of individuals who live in a multi-unit structure, 12) number of mobile homes, 13) number of crowded households (defined as having more people than rooms), 14) number of households with no vehicles, and 15) number of individuals living in group quarters.

Following SVI construction procedures (16), Orleans’ census tracts were ranked from lowest to highest in each of these 15 variables except for *per capita* income, which was ranked from highest to lowest as a higher income is indicative of lower vulnerability. After, a percentile rank was calculated for each of the 15 variables for each census tract, estimated as follows: *Percentile rank = (rank – 1)/(N-1)*. Next, each of the 15 variable’s percentile rank were summed to create an overall score. To rank census tracts based on their overall scores, an overall percentile rank was calculated following the same formula, with the final SVI ranking score ranging from 0.0 (less vulnerable) to 1.0 (most vulnerable).

### 2.1. Statistical analyses

Descriptive statistics were used to characterize the school meal distribution operations. For each pick-up location, the following characteristics were summarized: average meals available (weekly), average meals served (weekly), number of weeks of operation, and rate of meal pick-up ([average meals served/average meals available] x 100%).

In order to visualize the distribution of the meal sites in relation to New Orleans’ socioeconomic characteristics, we created three maps using the software QGIS version 3.28.3, overlaying the SVI ranking score categorized in quartiles and the 1) average meals available, 2) average meals served, and 3) rate of meal pick-up at each meal pick-up location; all three maps provided a similar visual story, so only the map presenting weekly average meals served is presented in this Brief Research Report (the other two maps are available upon request).

Further, we ran Pearson correlations between the SVI ranking score (continuous) and each of the meal distribution characteristics (average meals available, average meals served, number of weeks of operation, and rate of meal pick-up). Lastly, using the SVI ranking score categorized in quartiles (ranging from rank 1 = least socially vulnerable to rank 4 = most socially vulnerable), we ran an Analysis of Variance (ANOVA) analysis to examine the mean differences in average meals available, average meals served, number of weeks of operation, and rate of meal pick-up by SVI ranking score categories, using Tukey’s test as a post-hoc analysis where needed. All statistical analyses were conducted in SAS v9.4, with a value of *p* <0.05 denoting statistical significance.

## 3. Results

Based on data from NOLA Public Schools, 45 school meal pick-up sites were set up throughout the city in the immediate aftermath of the COVID-19 lockdowns (March 16 – May 31, 2020 – covering the school year since the start of lockdown). However, seven sites in the dataset had rates of meal pick-up that were greater than 100%, suggesting that more meals were served than were available; since we were unable to trace the source of the error in this accounting, these 7 pick-up sites were excluded from the sample for all analyses. From the remaining 38 sites, a total of 884,929 meals were available for pick-up during the 11-week observation period. On average, 2,405 meals were available weekly per site during the study period (range 496–6,116), with an average of 2,183 meals picked up weekly (range 293–5,253), yielding an average meal pick-up rate (picked-up vs. available meals) of 90.1% (range 59–100%). On average, pick-up sites were open for meal distribution for 9.5 weeks (range 0–11 weeks).

**Figure 1:**
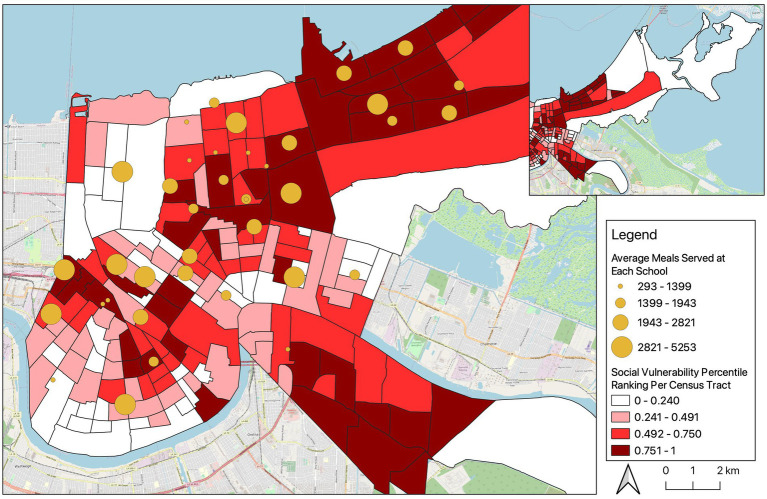
Location of school meals’ pick-up sites in New Orleans (March 16 – May 31, 2020), average weekly meals available per site, and census tract’s Social Vulnerability Index (SVI) ranking score categories.

Figure 1 visually depicts the location of school meals’ pick-up sites, the average weekly meals served per site, and each census tract’s SVI ranking score categories. We observe that pick-up sites were distributed throughout the city but located mostly in moderately (red, *n* = 14) or highly (maroon, *n* = 14) socially vulnerable areas (SVI > 0.492), with the exception of the Algiers/Old Aurora region, in the southern tip of the map, which appears to have only one pick-up site. It is important to note, however, that three of the seven pick-up sites excluded from the sample were located in this area (data not shown), suggesting adequate coverage.

There were weak and not statistically-significant correlations between SVI ranking (continuous) and average meals available, average meals served, number of weeks of operation, and rate of meal pick-up (Table 1). We also observed no significant differences in means of average meals available, average meals served, and number of weeks of operation by SVI rank (Table 2). However, descriptively, we observe that average meals available and average meals served were highest in census tracts ranked 2 in the SVI score (pink) and lowest in those ranked 3 (red). There was a statistically significant difference in the rate of meal pick-up, particularly between sites in census tracts ranked 2 (79.6% pick-up rate) compared to sites in census tracts ranked 3 (92.6% pick-up rate) in the SVI score (Table 2). Descriptively, rates of meal pick-up were highest in the most socially vulnerable census tracts (i.e., those ranked 3 and 4), compared to the least socially vulnerable tracts (ranked 1 and 2) (Table 2).

**Table 1:** Correlations between school meal variables and their corresponding census tract’s Social Vulnerability Index (SVI), *n* = 38 sites.

		Average meals available (weekly)	Average meals served (weekly)	Number of weeks of operation	Rate of pick-up (weekly)
SVI ranking	Pearson correlation value of *p*	−0.21330 0.1985	−0.17307 0.2988	0.05183 0.7573	0.28071 0.0878
Average meals available (weekly)	Pearson correlation value of *p*		**0.98421** **<0.0001**	0.30314 0.0643	0.14267 0.3928
Average meals served (weekly)	Pearson correlation value of *p*			**0.33235** **0.0415**	0.27984 0.0888
Number of weeks of operation	Pearson correlation value of *p*				**0.44386** **0.0052**

Bold values denote statistical significance at *p* < 0.05.

**Table 2:** Means of average meals available weekly, average meals served weekly, number of weeks of operation, and rate of pick-up weekly by Social Vulnerability Index (SVI) ranking; *n* = 38 sites.

	Rank 1 (white) (*n* = 6) Mean (SD)	Rank 2 (pink) (*n* = 4) Mean (SD)	Rank 3 (red) (*n* = 14) Mean (SD)	Rank 4 (maroon) (*n* = 14) Mean (SD)	*p*-value
Average meals available (weekly)	2669.5 (1894.1)	2917.8 (1732.1)	2016.6 (872.2)	2520.0 (1069.8)	0.4773
Average meals served (weekly)	2400.3 (1668.1)	2525.1 (1766.6)	1856.8 (783.1)	2318.4 (1019.9)	0.5992
Number of weeks of operation	9.5 (3.7)	9.3 (3.5)	8.9 (4.1)	10.2 (2.4)	0.7729
Rate of pick-up (weekly)	87.6 (10.9)	79.6 (18.7)^a^	92.6 (5.3)^a^	91.9 (5.1)	0.0459

The colors in each SVI rank category correspond to the colors in Figure 1. *p*-values from ANOVA.

a Means significantly different from each other based on Tukey’s post-hoc test.

## 4. Discussion

This Brief Research Report summarizes the school meal distribution operations carried out in New Orleans in the immediate aftermath of the COVID-19 triggered lockdowns. We found that NOLA Public Schools operated 45 meal pick-up sites throughout the city between March 16 and May 31, 2020, with reliable data on 38 of these 45 pick-up sites. Average number of meals available and served weekly were 2,405 and 2,183, respectively, with an average pick-up rate of 90%. Almost 37% of the pick-up sites (14 out of 38) were located in the most socially vulnerable neighborhoods, with an additional 37% located in moderately socially vulnerable neighborhoods. Neighborhood social vulnerability was not significantly associated with average meals available, average meals served, nor number of weeks of operation, though our small sample size may have limited statistical power to detect any differences. Neighborhood social vulnerability was associated with rate of meal pick-up, with statistically significant differences among census tracts ranked 2 (~80% pick-up rate) and 3 (~93% pick-up rate) in a 4-category vulnerability ranking, with 1 being the least vulnerable and 4 the most vulnerable.

Since New Orleans has among the highest rates of child poverty (8) and child food insecurity (9) in the country, our finding that three quarters of meal pick-up sites were located in the top two most socially vulnerable neighborhoods is an important one, suggesting the COVID-19 meal distribution operations were accessible to those most in need, despite the disaggregated nature of the charter school system. Previous research suggests that school meal programs – in particular the National School Lunch Program – reduce food insecurity among households with children (17). Specific to the COVID-19 pandemic and using a nationally representative sample, Morales et al. (18) found that having children enrolled in schools during October–November 2020 had no impact on household food insufficiency – a proxy for food insecurity. However, only 17% of the sample reported receiving food from schools (18), a much lower rate of meal pick-up than the one observed in this study.

Other research has also described COVID-19 related school meal distributions, particularly immediately after the lockdown declarations in March 2020 (19–24). In a study comparing COVID-related school meal distribution operations in four large school districts (Chicago, New York City, Los Angeles, and Houston), McLoughlin et al. (20) reported that most meal distribution sites were located in high-poverty areas, ranging from 62% of sites in Chicago, 67% in Los Angeles and New York City, and 68% in Houston. Similarly, most sites were in areas with a greater proportion of racial/ethnic minority households (60–76% of sites), with the exception of Houston, where only 40% of meal pick-up sites were located in such neighborhoods (20). Another study evaluating the Maryland COVID-19 school meal response found that most meal pick-up sites were located in areas with greater rates of child poverty (21). We also found that the majority (~75%) of meal pick-up sites in New Orleans were located in moderately/highly socially vulnerable neighborhoods. Further comparisons with previous studies are difficult given the different types of data used, methodologies employed, and geographies included (district vs. city vs. state), plus the uniqueness of New Orleans in terms of operating a city-wide charter school system. However, it is important to note that all studies have highlighted the importance of continuing to provide food to children during emergencies, even when implementation barriers exist (22).

This study has strengths and limitations. Strengths include the detailed operations data available, including the addresses of the meal distribution sites, allowing us to map the sites and evaluate geographic reach. Furthermore, we used a social vulnerability index – a more comprehensive way of assessing neighborhood demographic and socioeconomic characteristics than, for example, just looking at poverty rates or racial/ethnic minority make-up – to assess meal coverage from an equity perspective. As for limitations, there is no available information on who picked up the meals and who consumed the meals. It is possible that some community members without children picked up meals, and that meals picked up by parents or guardians of children were consumed by family members other than the target children. Further, the operations data did not include information on the nutrition composition of meals, so diet quality and nutrient adequacy could not be assessed. Moreover, we only had reliable operations data on 38 pick-up sites, which limited our statistical power for detecting associations between meal operations data and neighborhood characteristics. Finally, while parents/caregivers could pick up meals for their children in the pick-up site closest to their home, this may have been on a different census tract than their residence; thus, our results linking the operations’ data with neighborhoods’ social vulnerability should be interpreted with this caveat in mind.

In conclusion, NOLA Public Schools successfully pivoted to providing children with take-home meals due to COVID-19 lockdowns as of March 16, 2020, setting up 45 meal pick-up sites throughout the city, with three quarters of the sites assessed located in socially vulnerable neighborhoods. Future studies should focus on describing the types of meals provided to students during the COVID-19 lockdowns – in terms of quantity, quality, and nutrient adequacy – as well as on compiling “lessons learned” of what worked and did not work for future emergency preparedness. This is particularly important in a city like New Orleans, which is frequently affected by environmental disasters (e.g., hurricanes) that affect school operations.

## Data availability statement

The data analyzed in this study is subject to the following licenses/restrictions: data are not publicly available but were shared by NOLA Public Schools upon request by the authors. Requests to access these datasets should be directed to https://nolapublicschools.com/contact.

## Author contributions

MC, KC, and MK conceptualized the manuscript idea. MC obtained the data. JF, NE, and TN analyzed the data. MC and MK supervised the data analysis. MC wrote the first draft with contributions from JF. All authors contributed to the article and approved the submitted version.

## Funding

This research was supported in part by the Kellogg Foundation (Grant number P-6005931-2021) and by the National Institute of General Medical Sciences of the National Institutes of Health under Award Number TL4GM118968. The funders were not involved in the study design, collection, analysis, interpretation of data, the writing of this article or the decision to submit it for publication. The content is solely the responsibility of the authors and does not necessarily represent the official views of the National Institutes of Health.

## Conflict of interest

The authors declare that the research was conducted in the absence of any commercial or financial relationships that could be construed as a potential conflict of interest.

## Publisher’s note

All claims expressed in this article are solely those of the authors and do not necessarily represent those of their affiliated organizations, or those of the publisher, the editors and the reviewers. Any product that may be evaluated in this article, or claim that may be made by its manufacturer, is not guaranteed or endorsed by the publisher.
